# Complete mitochondrial genome of *Guangxia longlina* (Orthoptera: Tettigoniidae: Meconematinae)

**DOI:** 10.1128/mra.01169-25

**Published:** 2026-04-02

**Authors:** Ziyi Li, Yuqing Yao, Xun Bian, Bin Zhang

**Affiliations:** 1 College of Life Sciences & Technology, Inner Mongolia Normal University 71203 , Hohhot, China; 2 Key Laboratory of Ecology of Rare and Endangered Species and Environmental Protection (Guangxi Normal University), Ministry of Education 12388, Guilin, China; 3 Key Laboratory of Biodiversity Conservation and Sustainable Utilization for College and University of Inner Mongolia Autonomous Region, Hohhot, China; University of Maryland School of Medicine, Baltimore, Maryland, USA

**Keywords:** mitogenome, *Guangxia*, Meconematinae

## Abstract

We present the complete mitochondrial genome of *Guangxia longlina* based on a specimen from Tianlin, China. The mitogenome is 17,915 bp in length and is AT rich (69%). It consists of 13 protein-coding, 22 transfer RNA, and two ribosomal RNA genes and is identical in gene content to *Phlugiolopsis tuberculata*.

## ANNOUNCEMENT

Meconematinae is a diverse group of insects that is mainly distributed in Asia, Australia, and the Pacific Islands. In China, little is known about the mitochondrial genomes of the subfamily Meconematinae (1). *Guangxia longlina* An, Chen & Shi, 2023 was first reported from Guangxi, China (2), and resembles *Aphlugiolopsis* but differs in the concave posterior margin of the male 10th abdominal tergite with paired posterior processes and in styli positioned on the apex of the subgenital plate (3). To enrich molecular data and contribute to the phylogeny of *Guangxia*, the complete mitochondrial genome of *G. longlina* was assembled and annotated.

The specimen of *G. longlina*, an adult male collected on 16 September 2022, was collected in Tianlin, Guangxi, China (24.2711 N, 106.2058 E, 1,290 m), and the voucher is deposited at the collection of Guangxi Normal University. The DNA was extracted from the hind femur using the TIANamp Genomic DNA Kit. A 150 bp paired-end library was prepared using the MGIEasy Kit and subsequently sequenced on an Illumina NovaSeq 6000 platform (Illumina Inc.). The raw data were processed using fastp v0.20.0 (4) by trimming adapters and primers, filtering reads with phred quality <Q5, and filtering reads with N base number >3. The sequencing generated 31,496,800 reads that were filtered. Sequencing was performed by Beijing Berry Genomics Co., Ltd. The sequenced mitogenome data were assembled by NOVOPlasty v4.3.5 (5), generating a complete circular mitochondrial genome with an N50 of 17,915 and a GC content of 31%. Assembly was conducted under the following settings: assembly type = mito, genome size range = 14,000–18,000 bp, k-mer = 39, and extended log = 0. The mitochondrial genome of *Phlugiolopsis tuberculata* Xia & Liu, 1993, was identified as the closest reference through BLAST searches (6). Genome annotation was carried out via the MITOS2.1.9 web server (7), and initiation and termination codons were verified according to the invertebrate mitochondrial genetic code (8, 9). Nucleotide identities were calculated by BLAST search using the default settings. The mitochondrial genome map was visualized using Chloroplot version 0.2.4 (10).

**Fig 1:**
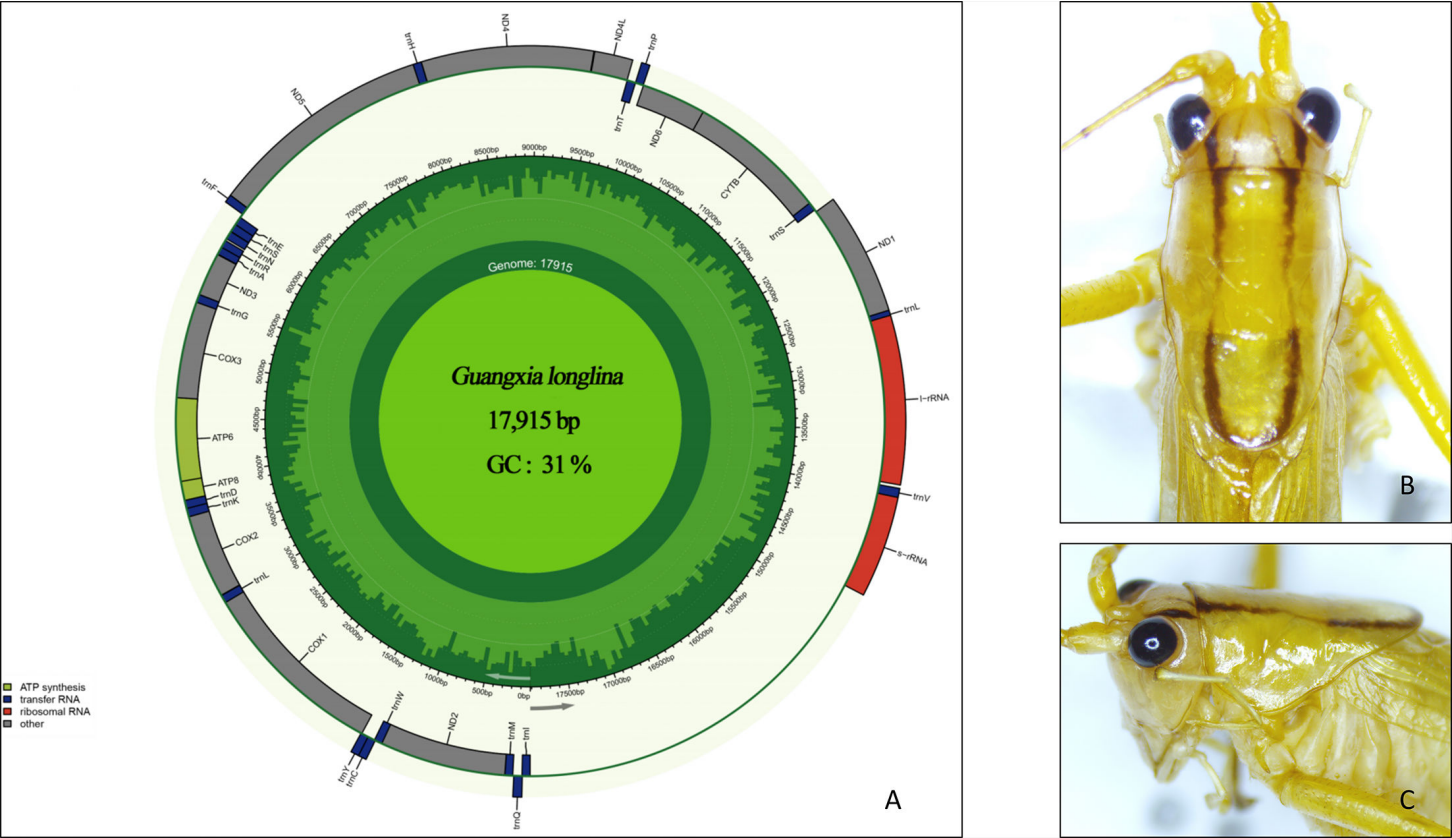
(**A**) Genome annotation was performed with MITOS and visualized using Chloroplot (12). The inner ring shows GC content (green) and transcription direction (arrows); the outer ring displays genes, with clockwise transcription inside and counterclockwise outside. Colors indicate gene groups as shown in the lower-left key. (**B**) Dorsal view of the head and pronotum of the adult male *G. longlina*. (**C**) Lateral view of the head and pronotum of the adult male *G. longlina*.

The complete circular mitochondrial genome of *G. longlina* is 17,915 bp in length and contains 37 genes, including 13 protein-coding genes, 22 transfer RNA genes, and 2 ribosomal RNA genes. The genome exhibits a strong AT bias, with an AT content of 69% (Fig. 1). Six of the protein-coding genes initiate with ATT (ATP8, COX1, ND2, ND3, ND4, and ND5), five with ATG (ATP6, COX2, COX3, ND4L, and cytochrome b [CYTB]), one with ATA (ND6), and one with TTG (ND1) (Table 1). Seven of the protein-coding genes terminate with TAA (ATP6, ATP8, COX1, ND2, ND3, ND4L, and ND6), and the T termination codon is found in the remaining five genes except CYTB and ND1 (TAG) (T stop codon is completed by the addition of 3′ A residues to the mRNA, as is common in animal mitochondrial genomes (11, 12).

**TABLE 1:** Mitochondrial genome content, organization, and codon information of *Guangxia longlina*

Gene	Type	Minimum nucleotide position	Maximum nucleotide position	Length	Start codon	Stop codon	Direction
tRNA-Ile	tRNA	1	67	67	–^*a*^	–	Forward
tRNA-Gln	tRNA	64	133	70	–	–	Reverse
tRNA-Met	tRNA	141	205	65	–	–	Forward
ND2	CDS	206	1234	1,029	ATT	TAA	Forward
tRNA-Trp	tRNA	1232	1298	67	–	–	Forward
tRNA-Cys	tRNA	1290	1355	66	–	–	Reverse
tRNA-Tyr	tRNA	1355	1419	65	–	–	Reverse
COX1	CDS	1412	2956	1,545	ATT	TAA	Forward
tRNA-Leu1	tRNA	2951	3016	66	–	–	Forward
COX2	CDS	3019	3703	685	ATG	T	Forward
tRNA-Lys	tRNA	3703	3773	71	–	–	Forward
tRNA-Asp	tRNA	3772	3838	67	–	–	Forward
ATP8	CDS	3839	4000	162	ATT	TAA	Forward
ATP6	CDS	3994	4671	678	ATG	TAA	Forward
COX3	CDS	4671	5457	787	ATG	T	Forward
tRNA-Gly	tRNA	5457	5522	66	–	–	Forward
ND3	CDS	5523	5876	354	ATT	TAA	Forward
tRNA-Ala	tRNA	5880	5944	65	–	–	Forward
tRNA-Arg	tRNA	5943	6006	64	–	–	Forward
tRNA-Asn	tRNA	6022	6089	68	–	–	Forward
tRNA-Ser1	tRNA	6091	6158	68	–	–	Forward
tRNA-Glu	tRNA	6158	6227	70	–	–	Forward
tRNA-Phe	tRNA	6258	6321	64	–	–	Reverse
ND5	CDS	6322	8053	1,732	ATT	T	Reverse
tRNA-His	tRNA	8053	8117	65	–	–	Reverse
ND4	CDS	8118	9444	1,327	ATT	T	Reverse
ND4L	CDS	9450	9746	297	ATG	TAA	Reverse
tRNA-Thr	tRNA	9747	9817	71	–	–	Forward
tRNA-Pro	tRNA	9816	9882	67	–	–	Reverse
ND6	CDS	9884	10411	528	ATA	TAA	Forward
CYTB	CDS	10411	11547	1,137	ATG	TAG	Forward
tRNA-Ser2	tRNA	11545	11614	70	–	–	Forward
ND1	CDS	11632	12579	948	TTG	TAG	Reverse
tRNA-Leu2	tRNA	12579	12644	66	–	–	Reverse
16S rRNA	rRNA	12619	13921	1,303	–	–	Reverse
tRNA-Val	tRNA	13947	14018	72	–	–	Reverse
12S rRNA	rRNA	14017	14805	789	–	–	Reverse

^*a*^ –, not applicable.

## Data Availability

The complete mitochondrial genome sequence of *Guangxia longlina* is available in GenBank under accession number PX412919. The associated BioProject, BioSample, and Sequence Read Archive (SRA) numbers are PRJNA1335651, SAMN52017803, and SRR35978746, respectively.
